# Optical Color Zero-Watermarking via Phase-Shifting Digital Holography Coupled with High-Robustness Bimodal Biometric Keys

**DOI:** 10.3390/e28090966

**Published:** 2026-08-29

**Authors:** Guanghai Liu, Zhe Zhang, Wang Fu, Cui Zhang, Boyu Wang, Yanfeng Su, Zhijian Cai

**Affiliations:** 1 College of Artificial Intelligence, Mianyang Teachers’ College, Mianyang 621000, China; zhezhang@mtc.edu.cn (Z.Z.); fwang@mtc.edu.cn (W.F.); cuizhang@mtc.edu.cn (C.Z.); 2 School of Opto-Electronical Engineering, Xi’an Technological University, Xi’an 710021, China; wangboyu@st.xatu.edu.cn; 3 College of Optical and Electronic Technology, China Jiliang University, Hangzhou 310018, China; 4 School of Optoelectronic Science and Engineering, Soochow University, Suzhou 215006, China; caizhijian@suda.edu.cn

**Keywords:** optical color zero-watermarking, digital holography, bimodal biometric keys, 3D face key, iris key

## Abstract

In this paper, an optical color zero-watermarking scheme based on robust bimodal biometric keys and phase-shifting digital holography is proposed. The color watermark is first encrypted into three amplitude ciphertexts through an optical encryption framework combining grating modulation, Fresnel-domain double random phase encoding (DRPE), and phase-shifting digital holography, where the phase masks are generated from biometric keys derived from the iris and three-dimensional (3D) face features of the encryption user. These high-level biometric features are extracted by a bimodal biometric high-order feature extraction network (BBHEN), including an iris high-order data extraction network and a 3D face high-order data extraction network. The extracted features of the color host image are then XORed with the corresponding ciphertexts, and the results are merged to construct a single zero-watermark image containing both host and watermark information. During extraction, biometric authentication is first performed to verify the identity of the decryption user. Only authorized users can recover the original watermark through zero-watermark reconstruction and extraction; otherwise, the process is terminated. Numerical simulations demonstrate the effectiveness, security, and robustness of the proposed scheme, particularly the strong protection capability of the bimodal biometric keys.

## 1. Introduction

In recent years, the protection of digital images has emerged as an important research topic in information security, owing to the increasing risks of unauthorized acquisition, malicious alteration, and improper distribution of sensitive image information. Among these technologies, image watermarking has attracted extensive attention from numerous research groups because of its favorable imperceptibility, excellent resistance to attacks, and reliable authentication capability [1,2]. Subsequently, various digital image watermarking methods based on the spatial domain [3,4,5], frequency domain [4,5,6,7], and optical principles have been successively proposed. Among them, optical image watermarking methods have received particularly widespread attention because of their high security, large key space, high degree of parallelism, and ability to process large-capacity image data [8,9]. Since the double random phase encoding (DRPE) technique was proposed by Refregier and Javidi in 1995 [10], numerous image watermarking methods based on DRPE have been developed. For example, Shao et al. [11] proposed a color image watermarking method based on DRPE in the gyrator domain. Bhnassy et al. [12] proposed an image watermarking method based on DRPE and chaotic system. Qin et al. [13] proposed an image watermarking method based on DRPE with sparse representation strategy. Thereafter, other advanced optical technologies, such as digital holography [14,15,16], computer generated holography (CGH) [17,18,19,20,21,22,23,24,25,26,27,28,29], ghost imaging [20,21,22], and ptychographic imaging [23,24], were gradually introduced into optical image watermarking methods. For example, Lee et al. [14] proposed an image watermarking method based on Fresnel diffraction and digital holography. Chen et al. [15] proposed a medical image watermarking method based on digital holography and three-level discrete wavelet transform-singular value decomposition (DWT-SVD). Yu et al. [16] proposed an image watermarking method based on digital holography and frequency localized contourlet transform (SFLCT). Subsequently, Wang et al. [17] proposed an image watermarking method based on CGH and discrete cosine transform. Zhu et al. [18] proposed a multiple-image watermarking method based on orbital angular momentum (OAM) holography and DWT-SVD. Zhu et al. [19] proposed a three-dimensional image watermarking method based on DWT-SVD and CGH under structured light illumination. On the other hand, Zhou et al. [20] proposed an image watermarking method based on ghost imaging and multiple logistic maps. Guan et al. [21] proposed a multiple-image encryption watermarking method based on ghost imaging and Arnold transform. Zhang et al. [22] proposed a zero watermarking method based on Zernike moments and correspondence ghost imaging. Furthermore, Su et al. [23] proposed a watermarking method based on single-pixel ptychography and variational image decomposition. Zhang et al. [24] proposed a watermarking method based on eccentric-rotation-scanned ptychography.

However, the aforementioned optical digital image watermarking methods generally have the problem of high key complexity, especially the random phase mask key is usually difficult to remember and carry. In addition, existing key mechanisms lack a reliable binding relationship with user identities. Once a key is lost or illegally stolen, sensitive information within the system can be easily obtained by unauthorized attackers, thereby seriously threatening the security of the encryption system. Biometric keys are considered an effective means of addressing the above problems because they offer the advantages of convenient portability, resistance to loss, and an inherent association with user identities. Therefore, in recent years, many research groups have attempted to introduce biometric keys into optical image watermarking methods and have conducted valuable research in this area [24,25,26,27,28,29,30,31,32]. For example, Su et al. [24] introduced a color image hiding encryption scheme by integrating fingerprint keys, phase-shifting digital holography, and watermarking techniques. Wang et al. [25] further developed a dual-image hiding encryption framework using fingerprint authentication and phase-shifting digital holography. Subsequently, several biometric-key-based image security approaches were reported. Wang et al. [26] investigated image watermarking using fingerprint keys and diffractive imaging, while Tao et al. [27] developed a palmprint-assisted image hiding encryption scheme combined with DWT-SVD. More recently, Su et al. [28] presented a face-key-driven blind watermarking method with a chaotic system, followed by zero-watermarking schemes based on face features and phase-shifting digital holography [29], phase-truncated fractional random transform with bimodal biometric keys [30], and diffractive imaging [31]. In addition, Wang et al. [32] proposed a three-dimensional image hiding encryption approach utilizing iris keys and computer-generated holography (CGH).

Although biometric keys have been successfully introduced into optical image watermarking methods, existing approaches still have several limitations. Some methods employ only a single biometric key, such as the iris, fingerprint, or palmprint, resulting in a relatively limited key space. Although bimodal biometric keys have also been investigated, their robustness remains insufficient. In particular, most face-key-based optical watermarking methods employ two-dimensional (2D) face images, whose limited feature information can be more easily intercepted, copied, or forged by unauthorized users. Moreover, slight facial rotation, expression changes, or occlusion during acquisition may cause considerable deviations in the extracted features, leading to authentication failure and termination of the decryption process. Here, an optical color zero-watermarking method based on highly robust bimodal biometric keys and phase-shifting digital holography is proposed. To the best of our knowledge, this is the first optical image watermarking method to introduce highly robust three-dimensional (3D) face and iris keys. During zero-watermark generation, the 3D face and iris images of the encryption user are processed by a bimodal biometric high-order feature extraction network (BBHEN), consisting of an iris high-order data extraction network (IHEN) and a 3D face high-order data extraction network (FHEN). The extracted high-order features are combined with chaotic mapping to generate bimodal biometric chaotic-structured light phase masks (BCSPMs). Meanwhile, the RGB channels of the color watermark are integrated through grating modulation and subsequently encoded using Fresnel-domain DRPE and phase-shifting digital holography to generate three amplitude-type ciphertexts. These ciphertexts are then combined with the Canny edge features extracted from the RGB channels of the host image through XOR operations and channel merging to generate the final zero-watermark image. During watermark extraction, the iris and 3D face data of the decryption user are processed by the BBHEN for bimodal biometric authentication. If authentication is successful, the color watermark is recovered through Canny edge feature extraction, digital holographic decoding, double random phase decoding (DRPD), and spatial filtering; otherwise, the decryption process is terminated. The use of high-order bimodal biometric features significantly improves the robustness of the biometric keys while maintaining their security. In addition, the generated zero-watermark image exhibits a noise-like distribution, effectively concealing the information of both the host and watermark images.

## 2. The Proposed Color Zero-Watermarking Method

### 2.1. Color Zero-Watermark Generation Process

**Figure 1:**
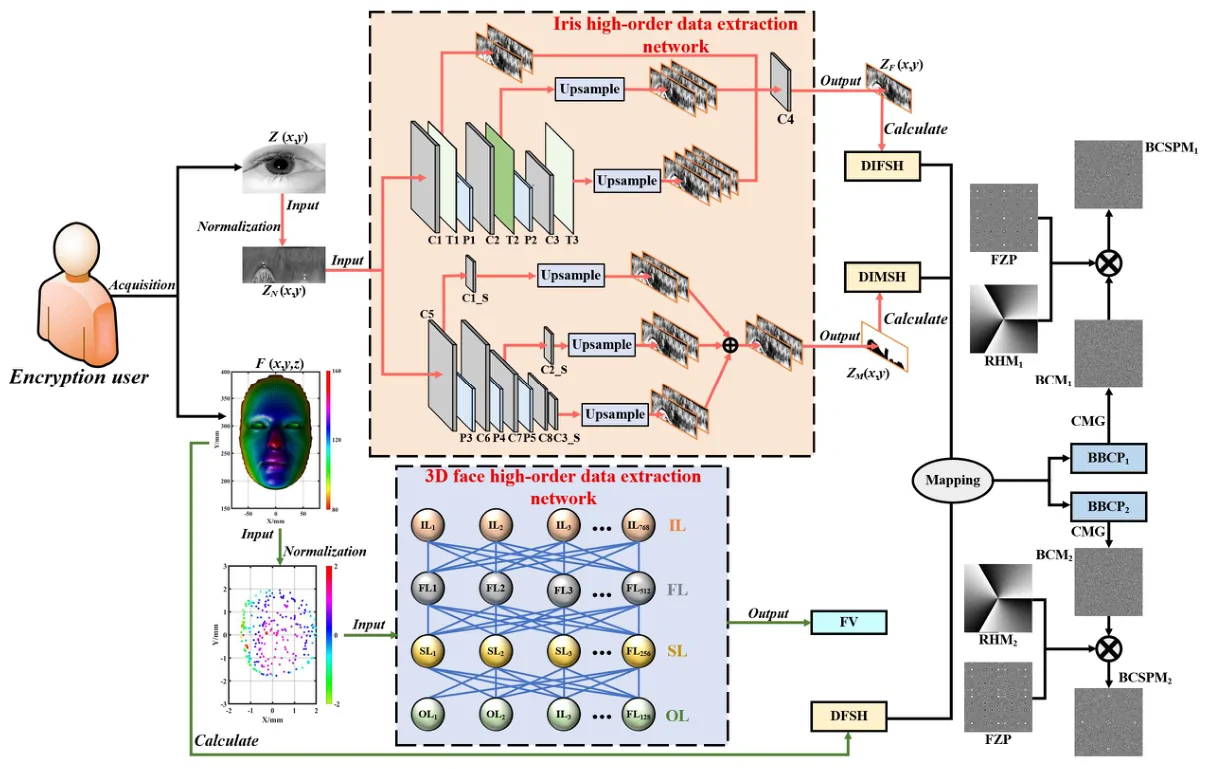
Framework diagrams of S1. CMG: chaotic matrix generation program.

**Figure 2:**
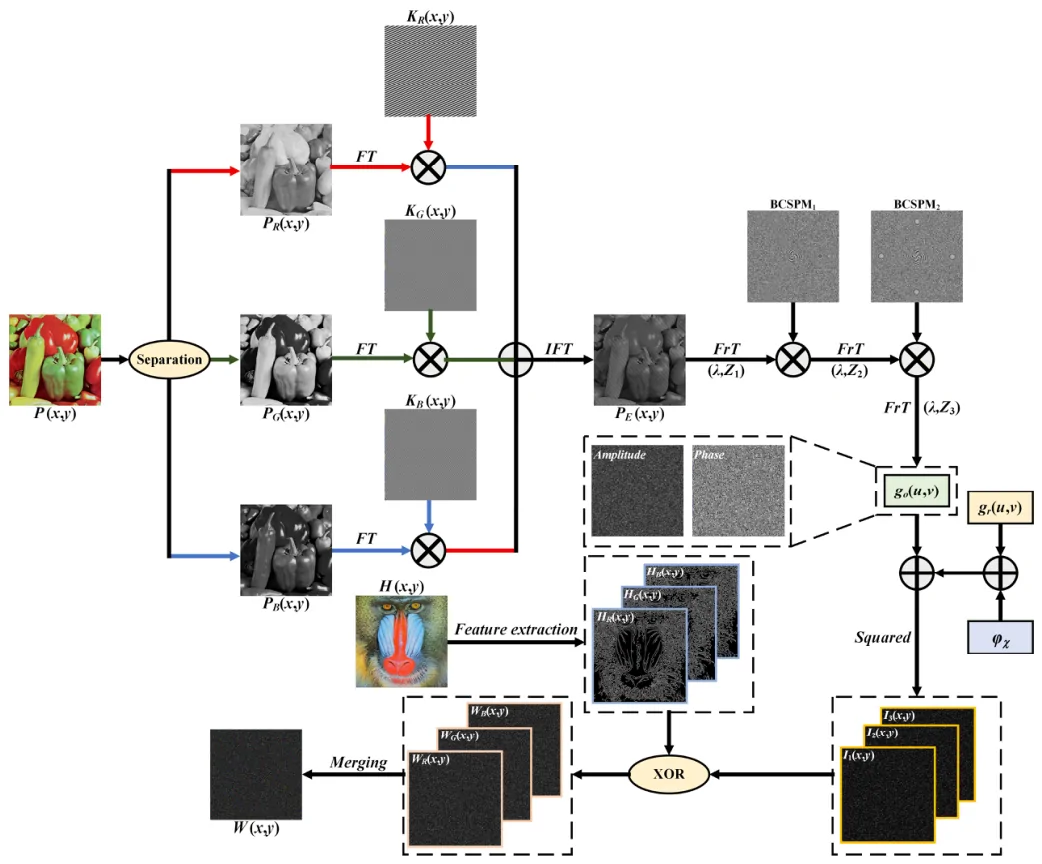
Framework diagrams of the S2.

The color zero-watermark generation procedure of the proposed method consists of two main stages: S1: Bimodal biological high-order data extraction and encryption key generation; S2: Color zero-watermark generation based on DRPE and phase-shift digital holographic coding. The detailed framework of S1 is illustrated in Figure 1, while the implementation flow of S2 is depicted in Figure 2.

S1: Bimodal biological high-order data extraction and encryption key generation can be divided into two steps:

(i) Bimodal biometric high-order feature acquisition: Firstly, the original 3D face image F(x,y,z) and iris image Z(x,y) of the encryption user are captured using the corresponding acquisition devices. Next, normalization operations are performed on F(x,y,z) and Z(x,y), respectively, thereby obtaining their corresponding normalized results $F_{N}(x,y,z)$ and $Z_{N}(x,y)$. Next, $F_{N}(x,y,z)$ and $Z_{N}(x,y)$ are input into a bimodal biometric high-order feature extraction network (BBHEN), which consists of an iris high-order data extraction network (IHEN) and a 3D face high-order data extraction network (FHEN), thereby obtaining the encryption user’s high-order iris feature $Z_{F}(x,y)$, high-order iris mask $Z_{M}(x,y)$, and high-order 3D face feature vector FV, which can be expressed as:
  (1) $$\left\{\begin{array}{l} Z_{F}(x,y),Z_{M}(x,y)=IHEN(Z_{N}(x,y))\\ FV=FHEN(F_{N}(x,y,z)),F_{N}(x,y,z))\in {\mathbb{R}}^{768},FV\in {\mathbb{R}}^{128} \end{array}\right.$$
  where the IHEN consists of one 3 × 7 convolutional layer (C1), one 3 × 5 convolutional layer (C2), six 3 × 3 convolutional layers (C3–C8), four 2 × 2 pooling layers (P1–P4), one 3 × 3 pooling layer (P5), three activation functions (T1–T3), and three 1 × 1 convolutional layers (C1_S–C3_S) [33,34]. Before feature extraction, the normalized iris image is resized to 64 × 512 pixels and subjected to contrast enhancement. In the original implementation, the network is trained using the Extended Triplet Loss (ETL), which incorporates iris masking and horizontal bit-shifting into the triplet-learning framework. Since the publicly released pretrained IHEN is directly adopted in the proposed method, the IHEN is not retrained in the present study, and the corresponding network architecture and original training mechanism are referred to Ref. [33]. The FHEN is trained in the present study using the FaceScape dataset. Each 3D face mesh is first normalized, and 256 vertices are retained. Since each vertex contains three spatial coordinates (*x*, *y*, *z*), each 3D face sample is converted into a 768-dimensional input vector. The FHEN feature extractor consists of three fully connected layers with 512, 256, and 128 neurons, respectively, and a ReLU activation function is employed after each fully connected layer. An additional fully connected classification layer is connected to the 128-dimensional feature vector during the training stage for subject classification. The FHEN is optimized using the cross-entropy loss function, which is expressed as:
  (2) $$L_{\mathrm{FHEN}}=-\frac{1}{N}\sum_{i=1}^{N}\sum_{c=1}^{C}\mu_{i,c}\log(\psi_{i,c})$$
  where *N* denotes the number of 3D face samples in a mini-batch, *C* denotes the number of subject classes, $\mu_{i,c}$ denotes the ground-truth label of the *i*-th sample, and $\psi_{i,c}$ denotes the predicted probability that the -th sample belongs to the *c*-th subject. By minimizing this loss function, the FHEN learns discriminative identity-related representations from the original 3D face data. The Adam optimizer is adopted with an initial learning rate of 1 × 10^−4^, and the batch size is set to 32. The FHEN is trained for a maximum of 100 epochs. The model achieving the highest validation accuracy is retained for subsequent feature extraction. In addition, random small-angle rotation, scaling, and Gaussian perturbation are employed as data-augmentation strategies to improve the generalization capability and robustness of the FHEN. After training, the classification layer is removed, and the 128-dimensional output of the third fully connected layer is retained for subsequent biometric authentication and key generation.
(ii) Bimodal biometric chaotic structured light phase masks (BCSPMs) generation: Firstly, the decimal hash value extraction technology is used to extract the decimal hash values of $Z_{F}(x,y)$, $Z_{M}(x,y)$, and F(x,y,z) respectively, and then the chaotic initial value mapping program is used to generate two sets of chaotic initial values, which can be expressed as
  (3) $$\left\{\begin{array}{l} IFSH^{M}={\left(\frac{1}{1+e^{IFSH}}\right)}^{M}\\ IMSH^{M}={\left(1-e^{-IMSH}\right)}^{M}\\ FSH^{M}={\left(\frac{2\arctan(FSM)}{\pi }\right)}^{M} \end{array}\right.$$
  where IFSH, IMSH and FSM are the corresponding decimal hash values of $Z_{F}(x,y)$, $Z_{M}(x,y)$ and F(x,y,z), respectively; M=1,2. Subsequently, $IFSH^{M}$, $IMSH^{M}$ and $FSH^{M}$ are input into the chaotic matrix program to generate Bimodal biometric chaotic matrices (BCM), where the general term $IFSH_{r+1}^{M}$ of the chaotic sequence can be expressed as
  (4) $$FSH_{n+1}^{M}=\frac{IMSF^{M}}{IFSH^{M}}\times FSH_{n}^{M}(1-{\left(FSH_{n}^{M}\right)}^{2})$$
  where $FSH_{1}^{M}={FSH}^{M}$ and M=1,2. Subsequently, the encryption user provides a set of digital keys, which are utilized to obtain the BCSPMs for the subsequent encryption operation via phase modulation. The corresponding expression is given as follows:
  (5) $$\begin{array}{cc} \;BCSPM_{M} & =\exp(j(BCM_{M}\times FZP\times RHM))\\ & =\exp(j(BCM_{M}\times \arg(\exp(-j\frac{\pi }{\lambda f}r^{2})\times \arg(\exp(j\rho 𝜛)))) \end{array}$$
  In this expression, r and $f_{M}$ represent the geometric radius and focal length parameters of the Fresnel zone plate (FZP). The variables $\rho_{M}$ and 𝜛 describe the topological charge number and spatial orientation angle associated with the radial Hilbert mask (RHM).

**Figure 3:**
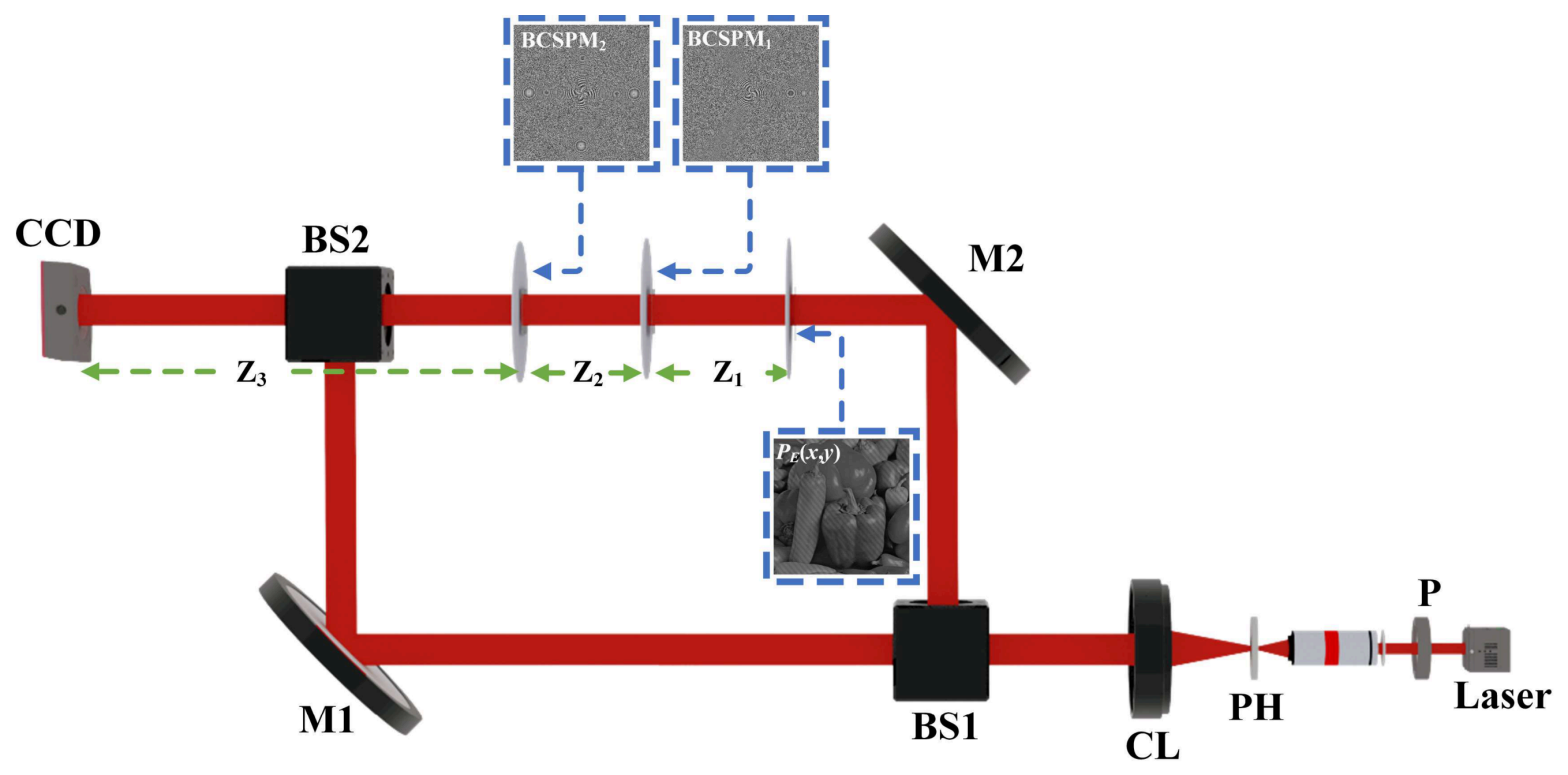
Optical setup corresponding to encryption step S2. The symbols P, OL, PH, CL, BS, L, and M indicate the polarizer, objective lens, pinhole, collimating lens, beam splitter, lens, and mirror, respectively.

S2: Color zero-watermark generation based on DRPE and phase-shift digital holographic coding, for which the optical path diagram is shown in Figure 3, can be divided into two steps:

(i) Ciphertext image generation: Firstly, the red channel data $P_{R}(x,y)$, green channel data $P_{G}(x,y)$ and blue channel data $P_{B}(x,y)$ of the original color watermark image P(x,y) are separated by channel separation technology. Subsequently, grating modulation is performed on $P_{R}(x,y)$, $P_{G}(x,y)$, and $P_{B}(x,y)$, so that the encryption fusion image $P_{E}(x,y)$ is generated, which can be expressed as
  (6) $$\;\;P_{E}(x,y)=\sum_{\psi =R}^{B}P_{\psi }(x,y)•K_{\psi }(x,y)$$
  where ψ=R,G,B, and $K_{\psi }(x,y)$ is the grating coefficient and its mathematical expression is denoted as:
  (7) $$\;K_{\psi }(x,y)=\left\{\frac{1}{2}+\frac{1}{2}\cos\left[2\pi f_{\psi }\left(x\cos\theta_{x\psi }+y\sin\theta_{y\psi }\right)\right]\right\}\mathrm{rect}\left(\frac{x}{X}\right)\mathrm{rect}\left(\frac{y}{Y}\right)$$
  where $f_{\psi }$, $\theta_{x\psi }$ and $\theta_{y\psi }$ describe the grating frequency and the horizontal/vertical inclination angles of the grating fringes, respectively, while X and Y define the dimensions of the grating. Then, the optical field $P_{E}(x,y)$ is propagated over a distance $Z_{1}$ using Fresnel diffraction at wavelength λ. The resulting field is subsequently encoded with $BCSPM_{1}$ Subsequently, the product undergoes a second Fresnel diffraction with the same wavelength and a propagation distance of $Z_{2}$, and the diffraction result is multiplied by $BCSPM_{2}$. Finally, the resulting product is subjected to a third Fresnel diffraction with the same wavelength and a propagation distance of $Z_{3}$, thereby generating the encryption object wave $g_{o}(u,v)$, which can be expressed as
  (8) $$g_{o}(u,v)=FrT_{\lambda ,Z_{3}}(FrT_{\lambda ,Z_{2}}(FrT_{\lambda ,Z_{1}}(P_{E}(x,y))\times BCSPM_{1})\times BCSPM_{2})$$
  where $FrT_{\lambda ,Z}(•)$ represents Fresnel diffraction with a wavelength of λ and a diffraction distance of Z. Secondly, the plane light wave with unit amplitude, which is coaxial with the $g_{o}(u,v)$, is introduced as a reference light wave $g_{r}(u,v)$. Subsequently, the phase shift $\varphi_{\chi }$ of the $g_{r}(u,v)$ is changed three times and makes $g_{r}(u,v)$ and $g_{o}(u,v)$ interfere in the recording plane, so that the three amplitude-type holographic ciphertexts $I_{\chi }(x,y)(\chi =1,2,3)$ are generated, which can be expressed as
  (9) $$\begin{array}{cc} I_{\chi }(u,v) & ={\left|g_{o}(u,v)+g_{r}(u,v)\exp\left(-j\phi_{\chi }\right)\right|}^{2}\\ & ={\left|g_{o}(u,v)\right|}^{2}+{\left|g_{r}(u,v)\right|}^{2}+g_{o}(u,v)g_{r}^{*}(u,v)\exp\left(j\phi_{\chi }\right)\\ & \;+g_{r}(u,v)g_{o}^{*}(u,v)\exp\left(-j\phi_{\chi }\right) \end{array}$$
  where $g_{o}(u,v)$ and $g_{r}(u,v)$ denote the encryption object wave and reference wave, respectively, and $\phi_{\chi }$ denotes the phase shift introduced into the reference wave, and χ=1,2,3. In the proposed method, the three phase shifts are explicitly set as $\phi_{1}=0,\;\phi_{2}=\pi /2,\;\phi_{3}=\pi$; $g_{r}{\left(u,v\right)}^{*}$ and $g_{o}{\left(u,v\right)}^{*}$ are denoted as conjugates of $g_{r}(u,v)$ and $g_{o}(u,v)$, respectively.
(ii) Color zero-watermark generation: Firstly, Canny edge detection is applied to the original color host image H(x,y) to obtain the features of its red, green, and blue channels ($H_{R}(x,y)$, $H_{G}(x,y)$, and $H_{B}(x,y)$). Subsequently, XOR operation is performed between $H_{R}(x,y)$, $H_{G}(x,y)$, $H_{B}(x,y)$, and $I_{1}(x,y)$, $I_{2}(x,y)$, $I_{3}(x,y)$, respectively, thereby generating the red channel data $W_{R}(x,y)$, green channel data $W_{G}(x,y)$, and blue channel data $W_{B}(x,y)$ of the color zero-watermark image W(x,y), which can be expressed as
  (10) $$\;\;\left\{\begin{array}{l} W_{R}(x,y)=H_{R}(x,y)⊕I_{1}(x,y)\\ W_{G}(x,y)=H_{G}(x,y)⊕I_{2}(x,y)\\ W_{B}(x,y)=H_{B}(x,y)⊕I_{3}(x,y) \end{array}\right.$$
  where ⊕ represents the XOR operation. Finally, the channel merging operation is performed on $W_{R}(x,y)$, $W_{G}(x,y)$, and $W_{B}(x,y)$, so that the final color zero-watermark image W(x,y) is generated. It is worth noting that the XOR operation used here is a pixel-wise bitwise XOR operation. Specifically, the holograms and Canny edge maps are converted into 16-bit grayscale images, where the binary values 0 and 1 in the edge maps are mapped to 0 and 65,535, respectively. Owing to the self-inverse property of XOR, the original holograms can be recovered by performing the same operation during extraction.

### 2.2. Zero-Watermark Extraction and Decoding Process

**Figure 4:**
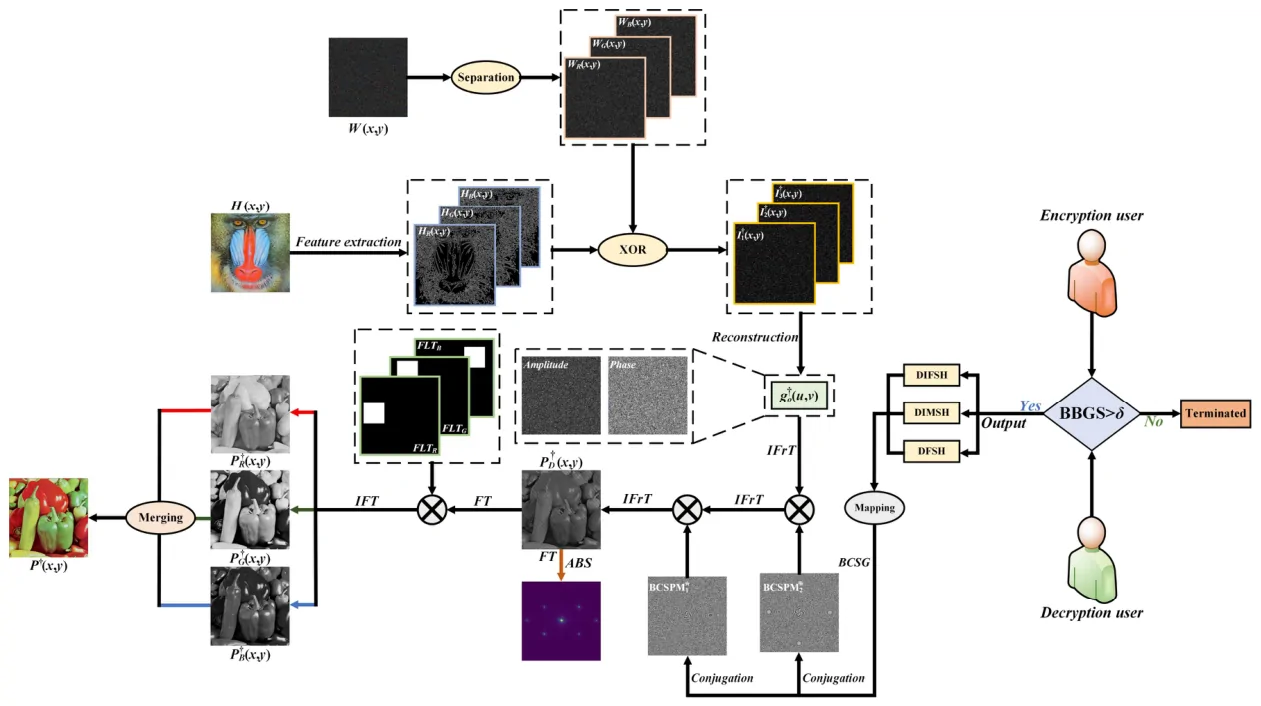
Framework diagrams of the J1-J2. BCSG: bimodal biometric chaotic structured light phase mask generation program.

The zero-watermark extraction and decoding process can be divided into two steps: J1: Biometric biometric authentication and decryption key acquisition; J2: Color watermark image reconstruction. The framework diagram for J1-J2 is shown in Figure 4.

J1: Biometric biometric authentication and decryption key acquisition can be divided into two steps:

(i) Biometric biometric authentication: Firstly, the original 3D face image $F^{†}(x,y,z)$ and iris image $Z^{†}(x,y)$ of the decryption user are captured using the corresponding acquisition devices. Subsequently, the same as the encryption step J1, the normalization operations are performed on $F^{†}(x,y,z)$ and $Z^{†}(x,y)$, respectively, thereby obtaining their corresponding normalized results $F_{N}^{†}(x,y,z)$ and $Z_{N}^{†}(x,y)$. Next, $F_{N}^{†}(x,y,z)$ and $Z_{N}^{†}(x,y)$ are input into a BBHEN, and thus the decryption user’s high-order iris feature $Z_{F}^{†}(x,y)$, high-order iris mask $Z_{M}^{†}(x,y)$, and high-order 3D face feature vector $FV^{†}$ are generated. Next, biometric authentication is performed on both the encryption user and the decryption user, thereby obtaining the bimodal biometric global similarity (BBGS) between the encryption user and the decryption user, which can be expressed as:
  (11)  BBGS=(IGS+FGS)/2
  where IGS and FGS denote the iris global similarity and 3D face global similarity, respectively. It should be noted that authentication is considered successful only when BBGS, IGS, and FGS all meet the preset threshold. Furthermore, the mathematical expressions of IGS and FGS are as follows:
  (12) $$\;\left\{\begin{array}{l} IGS=\frac{1}{1+HAM}\\ FGS=\frac{FV^{†}•FV}{\left\|FV^{†}\right\|\times \left\|FV\right\|} \end{array}\right.$$
  where *HMA* denotes the average of the Hamming distance between the $Z_{F}^{†}(x,y)$ and $Z_{M}^{†}(x,y)$ and that between the $Z_{F}(x,y)$ and $Z_{M}(x,y)$. Therefore, Equation (11) can be rewritten as follows:
  (13) $$\begin{array}{cc} \;BBGS & =(\frac{1}{1+HAM}+\frac{FV^{†}•FV}{\left\|FV^{†}\right\|\times \left\|FV\right\|})/2\\ & =\;\frac{(FV^{†}•FV)(1+HMA)+\left\|FV^{†}\right\|\times \left\|FV\right\|}{2\left[\left\|FV^{†}\right\|\times \left\|FV\right\|\right](1+HMA)} \end{array}$$
  If the BBGS exceeds a predefined threshold δ, authentication is successful, and the subsequent decryption procedure is initiated; otherwise, authentication fails, and the decryption process is terminated.
(ii) Decryption key acquisition: After successful authentication, the DIFSH, DIMSH, and DFSH stored in the system are retrieved, and the same operations as those in encryption step J1 are performed to generate the BCSPMs. Finally, the complex conjugates of the BCSPMs are calculated, thereby generating the decryption keys ($BCSPM^{*}$) required for the subsequent decryption step.

J2: Color watermark image reconstruction can be divided into two steps:

(i) Decryption hologram generation: Firstly, Canny edge detection is applied to the original color host image H(x,y) to obtain the features of its red channel data $H_{R}(x,y)$, green channel data $H_{G}(x,y)$, and blue channel data $H_{B}(x,y)$. Subsequently, XOR operation is performed between the $H_{R}(x,y)$, $H_{G}(x,y)$, $H_{B}(x,y)$ and the red channel data $W_{R}(x,y)$, green channel data $W_{G}(x,y)$, and blue channel data $W_{B}(x,y)$ of the color zero-watermark image W(x,y). Thus, the decryption hologram $I_{\chi }^{†}(x,y)(\chi =1,2,3)$ containing all the information of the original color watermarked image is generated, which can be expressed as
  (14) $$\;\;\left\{\begin{array}{l} I_{1}^{†}(x,y)=H_{R}(x,y)⊕W_{R}(x,y)\\ I_{2}^{†}(x,y)=H_{G}(x,y)⊕W_{G}(x,y)\\ I_{3}^{†}(x,y)=H_{B}(x,y)⊕W_{B}(x,y) \end{array}\right.$$
(ii) Decryption watermark image generation: Firstly, complex amplitude reconstruction is performed on the decryption hologram $I_{\chi }^{†}(x,y)(\chi =1,2,3)$ to obtain the decrypted complex amplitude $g_{o}^{†}(u,v)$, which can be expressed as
  (15) $$g_{o}^{†}(u,v)=\frac{\left(1+j)•(I_{1}^{†}(x,y)-I_{2}^{†}(x,y))+(j-1)•(I_{3}^{†}(x,y)-I_{2}^{†}(x,y)\right)}{4g_{r}^{*}(u,v)}$$
  Subsequently, $g_{o}^{†}(u,v)$ undergoes inverse Fresnel diffraction with a wavelength of $\lambda^{†}$ over a propagation distance of $Z_{3}^{†}$, and the resulting diffracted wave is multiplied by $BCSPM_{2}^{*}$. The product then undergoes inverse Fresnel diffraction with a wavelength of $\lambda^{†}$ over a propagation distance of $Z_{2}^{†}$, after which the resulting diffracted wave is multiplied by $BCSPM_{1}^{*}$. Finally, inverse Fresnel diffraction with a wavelength of $\lambda^{†}$ over a propagation distance of $Z_{1}^{†}$ is performed, and the amplitude of the resulting diffracted wave is extracted to obtain the decryption fusion image $P_{D}(x,y)$, which can be expressed as:
  (16) $$P_{D}^{†}(x,y)=IFrT_{\lambda ,Z_{1}^{†}}(IFrT_{\lambda ,Z_{2}^{†}}(IFrT_{\lambda ,Z_{3}^{†}}(g_{o}^{†}(u,v))\times BCSPH_{1}^{*}))\times BCSPH_{2}^{*})$$
  where $IFrT_{\lambda ,Z}(•)$ represents inverse Fresnel diffraction with a wavelength of λ and a diffraction distance of Z. Next, spatial filtering is performed on $P_{D}(x,y)$ to extract the red channel data $P_{R}^{†}(x,y)$, green channel data $P_{G}^{†}(x,y)$, and blue channel data $P_{B}^{†}(x,y)$ of the decryption watermark image $P^{†}(x,y)$, which can be expressed as
  (17) $$\;P_{\psi }(x,y)=IFT(FT(P_{D}(x,y))•FLT_{\psi })$$
  where $FLT_{\psi }$, FT(⋅) and IFT(⋅) represents the spatial filter, Fourier transform and inverse Fourier transform; ψ=R,G,B. Finally, $P_{R}^{†}(x,y)$, $P_{G}^{†}(x,y)$, $P_{B}^{†}(x,y)$ are performed as a channel merge operation, and the decryption watermark image $P^{†}(x,y)$ is generated.

## 3. Simulations and Results

### 3.1. Encryption and Decryption

To demonstrate the feasibility of the proposed color zero-watermarking method, a series of computational simulations were conducted using Python 3.9.0 and MATLAB 2021b.

**Figure 5:**
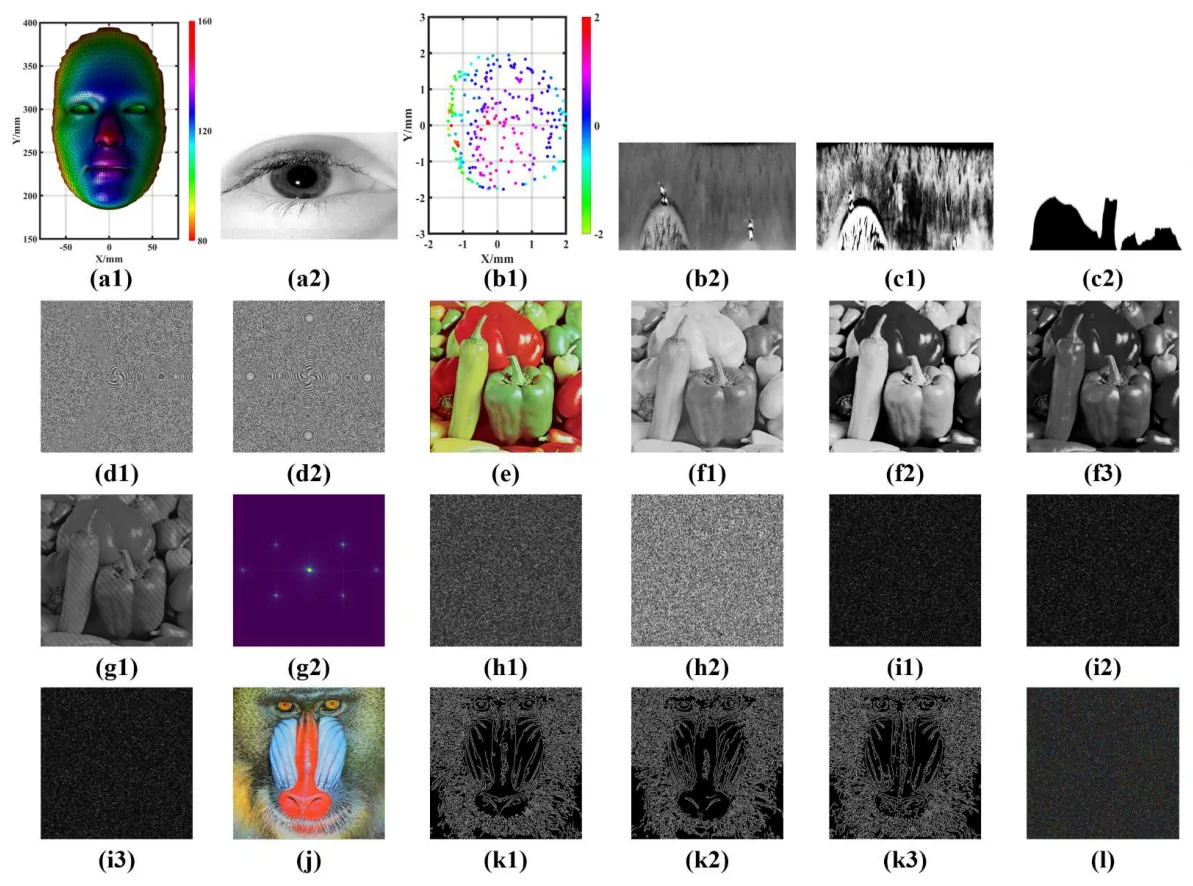
(**a1**,**a2**) the original 3D face image F(x,y,z) and iris image Z(x,y) of the encryption user; (**b1**,**b2**) the normalized results corresponding to (**a1**,**a2**); (**c1**,**c2**) the high-order iris feature $Z_{F}(x,y)$, high-order iris mask $Z_{M}(x,y)$ of Z(x,y); (**d1**,**d2**) the bimodal biometric chaotic structured light phase mask generation $BSPM_{1}$ and $BSPM_{2}$; (**e**) the original color watermark image P(x,y); (**f1**–**f3**) the red, green, and blue channel data corresponding to P(x,y); (**g1**,**g2**) the encryption fusion image and the spectrum of the (**g1**); (**h1**,**h2**) the amplitude and phase of $g_{o}(u,v)$; (**i1**–**i3**) the three amplitude type holographic ciphertexts $I_{\chi }(x,y)(\chi =1,2,3)$; (**j**) the host image H(x,y); (**k1**–**k3**) the feature maps of the H(x,y); (**l**) the final zero-watermark image W(x,y).

**Figure 6:**
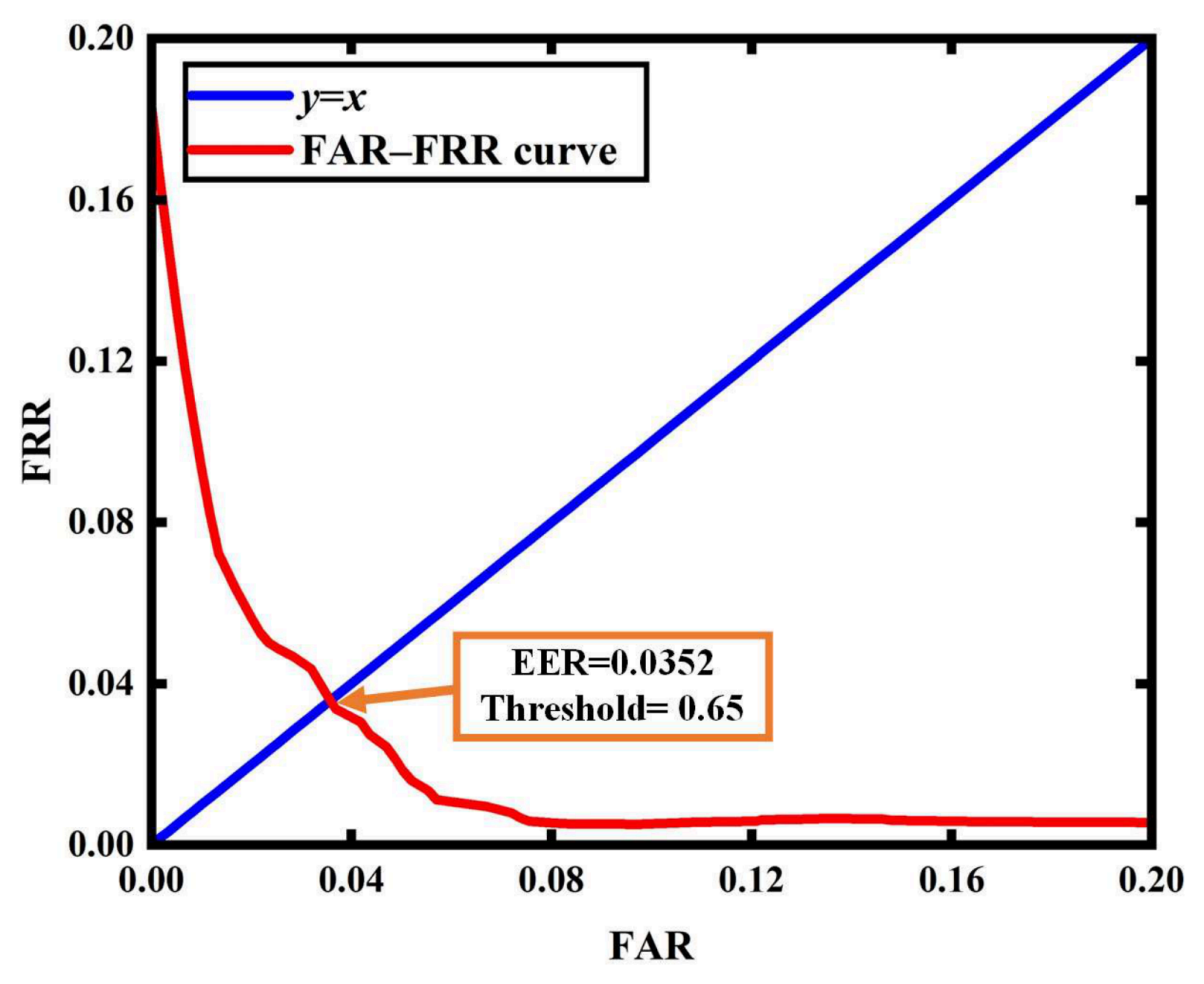
FAR–FRR curve of the BBGS.

Firstly, as shown in Figure 5(a1,a2), the original 3D face image F(x,y,z) and iris image Z(x,y) of the encryption user are captured using the corresponding acquisition devices. Subsequently, F(x,y,z) and Z(x,y) are normalized to obtain the corresponding normalized results $F_{N}(x,y,z)$ and $Z_{N}(x,y)$, as shown in Figure 5(b1,b2). Then, BBHEN is employed to extract the high-order information from F(x,y,z) and Z(x,y), where the high-order iris feature $Z_{F}(x,y)$, high-order iris mask $Z_{M}(x,y)$ of the Z(x,y) are illustrated in Figure 5(c1,c2), respectively. Subsequently, the bimodal biometric chaotic structured light phase mask generation program is utilized to generate $BSPM_{1}$ and $BSPM_{2}$, as shown in Figure 5(d1,d2). Next, the original color watermark image P(x,y) is selected from the USC-SIPI image database [35], as shown in Figure 5e. Moreover, the corresponding red, green, and blue channel data ($P_{R}(x,y)$, $P_{G}(x,y)$, and $P_{B}(x,y)$) components of P(x,y), are separately displayed in Figure 5(f1–f3). Subsequently, $P_{R}(x,y)$, $P_{G}(x,y)$, and $P_{B}(x,y)$ are individually subjected to grating modulation using three amplitude-type gratings, thereby generating the encryption fusion image $P_{E}(x,y)$ as shown in Figure 5(g1). In addition, the spectrum of the $P_{E}(x,y)$ is also presented in Figure 5(g2). Next, $BSPM_{1}$ and $BSPM_{2}$ are employed to perform DRPE encryption on P(x,y) generating the encryption object wave $g_{o}(u,v)$ in terms of amplitude and phase, as illustrated in Figure 5(h1,h2). The parameters of the first diffraction distance $Z_{1}$, the second diffraction distance $Z_{2}$, the third diffraction distance $Z_{3}$ and the wavelength λ are 140 mm, 150 mm, 160 mm and 632.8 nm, respectively. Subsequently, a phase-shift digital holographic coding is performed on $g_{o}(u,v)$ producing the three amplitude-type holographic ciphertexts $I_{\chi }(x,y)(\chi =1,2,3)$, as shown in Figure 5(i1–i3). Furthermore, Canny edge detection is applied to the host image H(x,y) shown in Figure 5j, generating the feature maps of its red, green, and blue channels, denoted as $H_{R}(x,y)$, $H_{G}(x,y)$, and $H_{B}(x,y)$, respectively, as shown in Figure 5(k1–k3). Finally, $I_{\chi }(x,y)(\chi =1,2,3)$ are individually XORed with $H_{R}(x,y)$, $H_{G}(x,y)$, and $H_{B}(x,y)$, respectively. The XOR results are then combined through an appropriate fusion operation, thereby generating the color zero-watermark image W(x,y), as shown in Figure 5l. It should be noted that all 3D face keys and iris keys used in this study are obtained from the FaceScape dataset [36] and the CASIA-IrisV4 dataset [37], respectively. To quantitatively determine the authentication threshold of the bimodal biometric global similarity (BBGS), an additional FAR–FRR analysis is performed. Specifically, the iris high-order data extraction network (IHEN) and the 3D face high-order data extraction network (FHEN) independently generate the authentication similarity scores for the iris and 3D face biometric keys, respectively, and the BBGS is calculated as the average of these two similarity scores. Accordingly, the FAR and FRR of the BBGS at each threshold are obtained by averaging the corresponding FAR and FRR values of the IHEN- and FHEN-based authentication systems. By gradually varying the authentication threshold, the FAR–FRR curve of the BBGS is obtained, as shown in Figure 6. The two curves intersect at approximately 0.0352, corresponding to an equal error rate (EER) of 0.0352. The BBGS threshold corresponding to this EER operating point is 0.65. This threshold provides a reasonable balance between false acceptance and false rejection rather than being arbitrarily assigned.

**Figure 7:**
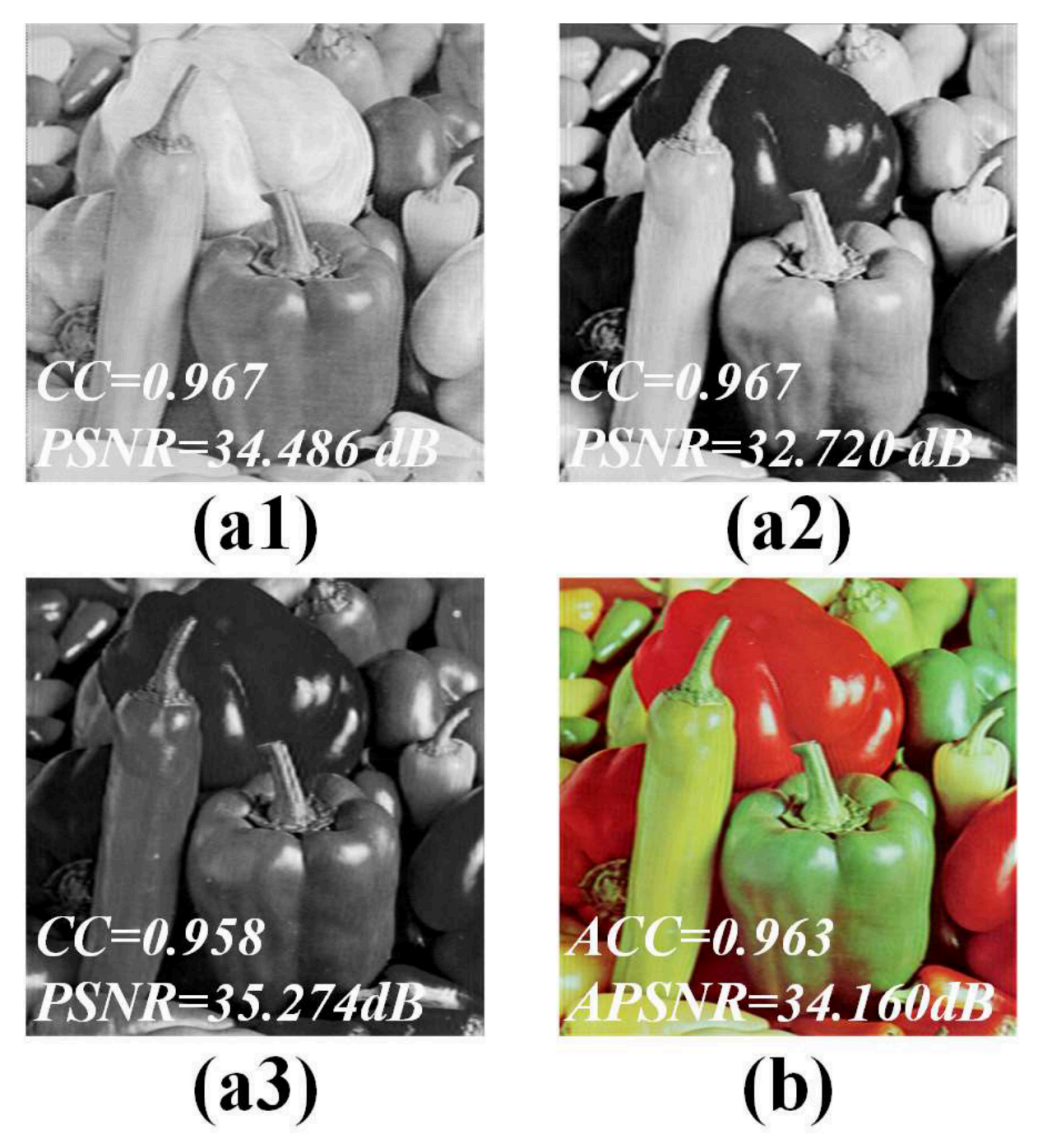
(**a1**–**a3**) Red channel data, green channel data, blue channel data of the final decryption watermark image, respectively; (**b**) the final decryption watermark image.

Subsequently, the zero-watermark reconstruction and extraction operations are carried out to verify whether the employed keys are valid. In this stage, the decryption user provides the 3D face key, iris key, and a set of optical parameters required for image recovery. The average correlation coefficient (ACC) and average PSNR (APSNR) are adopted to quantitatively characterize the similarity between the recovered and original images, and their mathematical expression is given as follows:

(18) $$\;\left\{\begin{array}{l} ACC=\frac{CC_{R}+CC_{G}+CC_{B}}{3}\\ APSNR=\frac{PSNR_{R}+PSNR_{G}+PSNR_{B}}{3} \end{array}\right.$$

where $CC_{𝜛}$ and $PSNR_{𝜛}$ represent the CC and PSNR value at 𝜛=R,G,B channel between the decryption result and the original image, which can be expressed as

(19) $$\;\left\{\begin{array}{l} {\mathrm{CC}}_{𝜛}=\frac{\left|\sum_{x=1}^{X}\sum_{y=1}^{Y}[P_{𝜛}(x,y)-\bar{P_{𝜛}}][P_{𝜛}^{†}(x,y)-\bar{P_{𝜛}^{†}}]\right|}{\sqrt{\left[\sum_{x=1}^{X}\sum_{y=1}^{Y}{\left(P_{𝜛}(x,y)-\bar{P_{𝜛}}\right)}^{2}\right]\left[\sum_{x=1}^{X}\sum_{y=1}^{Y}{\left(P_{𝜛}^{†}(x,y)-\bar{P_{𝜛}^{†}}\right)}^{2}\right]}}\\ PSNR_{𝜛}=10\log_{10}\left[\frac{256^{2}}{\frac{1}{XY}\sum_{x=1}^{X}\sum_{y=1}^{Y}{\left(P_{𝜛}(x,y)-P_{𝜛}^{†}(x,y)\right)}^{2}}\right] \end{array}\right.$$

where $\bar{P_{𝜛}}$ and $\bar{P_{𝜛}^{†}}$ respectively represent the pixel grayscale mean values of the plaintext image and the decrypted image at channel 𝜛=R,G,B. Figure 7(a1–a3) present the red, green, and blue channel components of the decrypted watermark image, respectively, when all keys are correct. Moreover, Figure 7b shows the final decryption watermark image. As shown in Figure 7, the CC and PSNR values corresponding to the reconstructed R, G, and B components are all higher than 0.95 and 32 dB, respectively. These results demonstrate that, although a small amount of information loss occurs during the spatial filtering process, the proposed method can still achieve high-quality reconstruction of the original color watermark image.

### 3.2. Security Analysis

**Figure 8:**
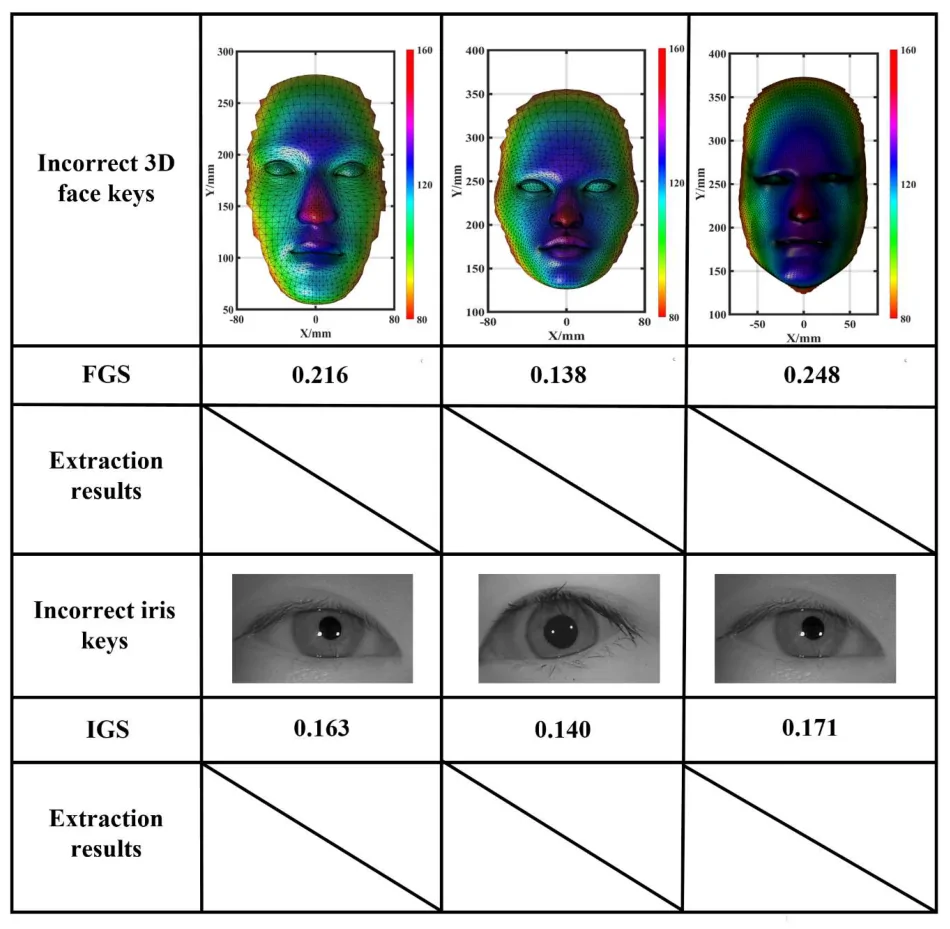
The extraction results when 3Dface key or iris key is incorrect.

Next, the performance and security characteristics of the bimodal biometric keys and digital key employed in the proposed scheme are investigated. Figure 8 shows the extraction results obtained with incorrect 3D face or iris keys provided by the decryption user. It is worth noting that the incorrect face keys or iris keys used in Figure 8 are randomly collected from more than 500 unauthorized attackers. Furthermore, it should be clarified that Figure 8 only presents the extraction results when either the 3D face key or the iris key alone is incorrect. The extraction results when both biometric keys are simultaneously incorrect are similar to those shown in Figure 8. Figure 8 demonstrates that the use of an incorrect 3D face key or iris key leads to a significant reduction in the FGS or IGS or BBGS between the decryption and encryption users. Once the FGS or IGS or BBGS value is lower than the predefined threshold (0.65), the user authentication is rejected, preventing further decryption.

**Figure 9:**
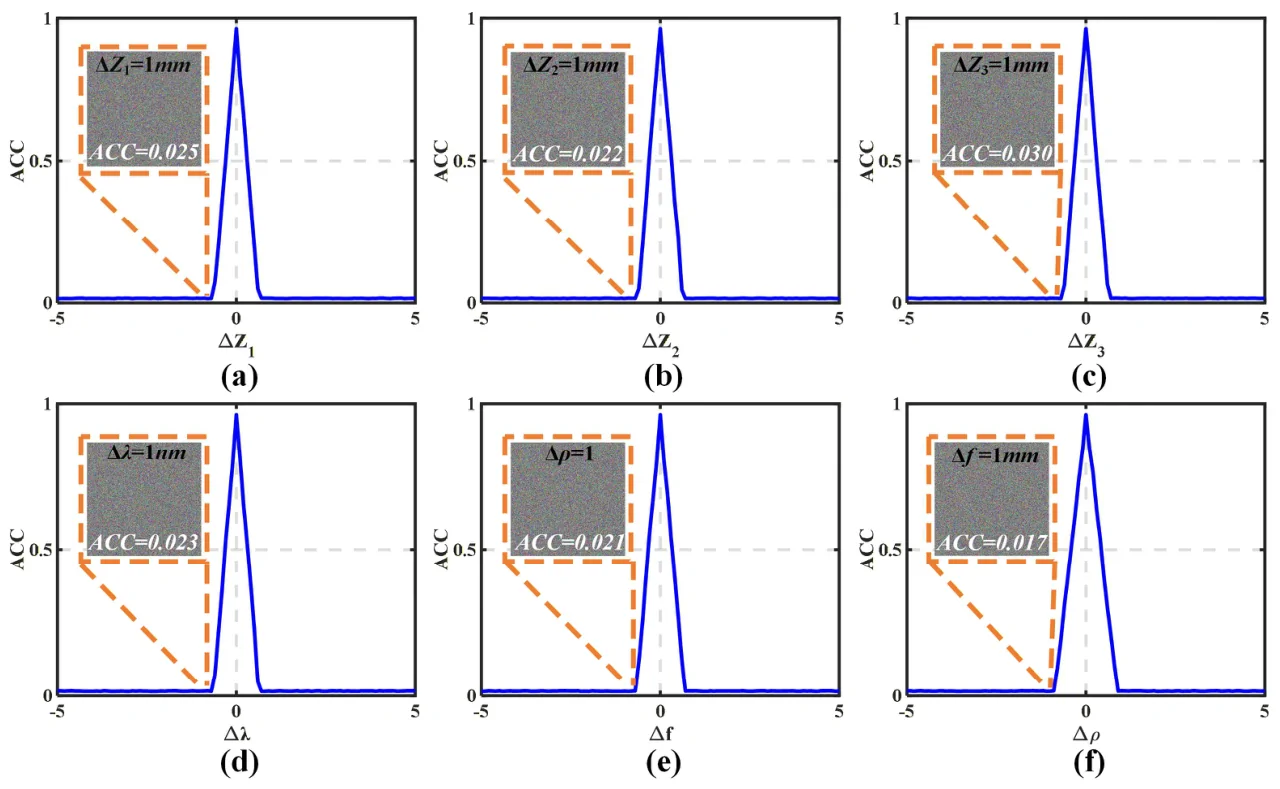
Sensitivity analysis of the digital keys, including (**a**) $Z_{1}$, (**b**) $Z_{2}$, (**c**) $Z_{3}$, (**d**) λ, (**e**) f, and (**f**) ρ.

Moreover, the influence of digital key perturbations on the proposed encryption system is evaluated. The sensitivity responses illustrated in Figure 9a–f correspond to the deviations of six key parameters, namely the three diffraction distances $\Delta Z_{1}$, $\Delta Z_{2}$, and $\Delta Z_{2}$), wavelength (Δf), focal length (Δf), and topological charge number (Δρ). Specifically, the deviations of the first, second, and third diffraction distances are introduced as $\Delta Z_{1}=1\;mm$, $\Delta Z_{2}=1\;mm$, and $\Delta Z_{3}=1\;mm$, respectively. Meanwhile, the deviations of the wavelength key, focal length key, and topological charge number key are introduced as Δλ=1 nm, Δf=1 mm, and Δρ=1, respectively. The corresponding extraction results under these key deviations are also marked in Figure 9a–f.

Furthermore, the key space of the proposed scheme is evaluated. The corresponding results for the digital keys are summarized in Table 1. Here, the sensitivity is defined as the minimum variation of each digital parameter that causes the ACC value to decrease to 0.1. Under this condition, the recovered image becomes noise-like and no recognizable plaintext information can be obtained. The parameter ranges listed in Table 1 are determined according to the positive range of integer-type data. Consequently, the overall key space provided by the digital keys is approximately 2^93^, demonstrating the strong resistance of the proposed method against exhaustive key search attacks. Moreover, the current SPM is generated using only two parameters, namely the topological charge and the focal length. Although such a relatively simple SPM is adopted in this work, spatial phase masks containing more adjustable parameters can be introduced in future studies, which would further increase the dimensionality of the key space and thereby enhance the security of the proposed method. Although the biometric features are not included in the cryptographic key-space calculation, the use of two independent biometric modalities, namely the 3D face and iris, strengthens identity authentication and increases the difficulty of unauthorized access to the decryption process.

It should be noted that, in this framework, the 3D face and iris features are used to verify user identity and control access to the decryption process. Since their effective entropy has not been quantitatively established, these biometric features are not treated as uniformly random cryptographic key bits and are therefore excluded from the key-space calculation. Although the bimodal biometric features do not contribute additional cryptographic key entropy, they establish an independent identity-verification barrier, thereby substantially increasing the cost and complexity for an unauthorized attacker to access or reconstruct sensitive watermark information.

### 3.3. Bimodal Biometric Key Robustness Analysis

Furthermore, the robustness of the bimodal biometric keys employed in the proposed method, which include the 3D face key and iris key, is also evaluated. It should first be clarified that, during the robustness tests of the iris key and 3D face key, the corresponding biometric similarity score was used as the evaluation metric. Specifically, when evaluating the robustness of the iris key, the iris global similarity (IGS) was used, whereas when evaluating the robustness of the 3D face key, the 3D face global similarity (FGS) was used. This setting prevents the high similarity score of the unattacked biometric key from compensating for a decrease in the similarity score of the attacked biometric key, thereby ensuring that the robustness of the iris key and 3D face key can be evaluated independently and accurately.

**Figure 10:**
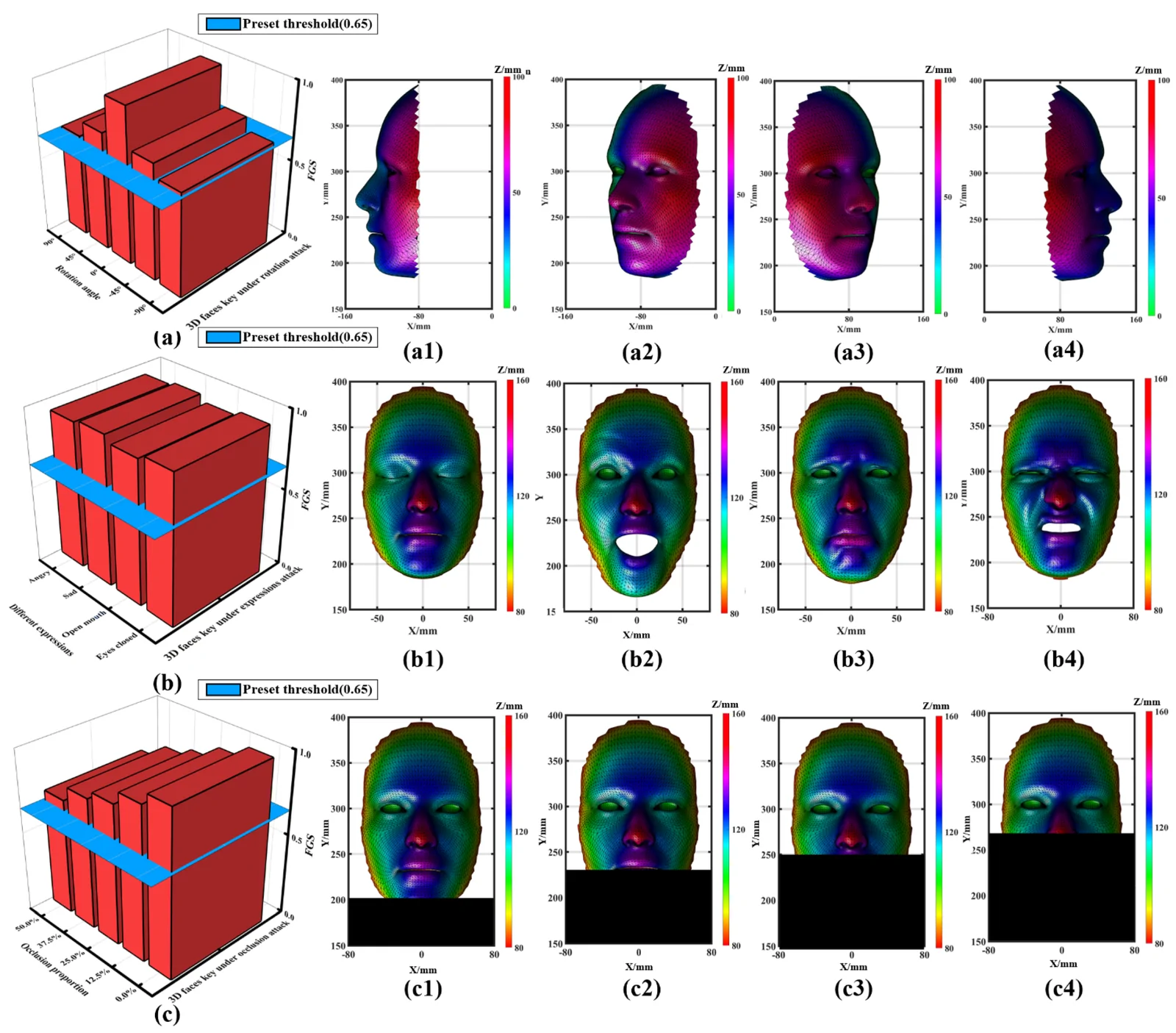
Robustness evaluation of the 3D face key used in the proposed scheme. (**a**–**c**) the mapping relationship between the 3D face key and FGS under rotation attacks, expression attacks, and occlusion attacks. (**a1**–**a4**) the 3D face key subjected to rotation attacks of −90°, −45°, 45°, and 90°, respectively. (**b1**–**b4**) the 3D face key subjected to expression attacks of eyes closed, open mouth, sad, and angry, respectively. (**c1**–**c4**) the 3D face key subjected to occlusion attacks of 12.5%, 25%, 37.5%, and 50%, respectively.

It should be noted that all 3D face keys and iris keys used in the following robustness analyses are obtained from the FaceScape dataset [36] and the CASIA-IrisV4 dataset [37], respectively. Firstly, Figure 10a–c illustrate the mapping relationship between the 3D face key and FGS under rotation attacks, expression attacks, and occlusion attacks. Figure 10(a1–a4) show the results of the 3D face key subjected to rotation attacks of −90°, −45°, 45°, and 90°, respectively. Figure 10(b1–b4) show the results of the 3D face key subjected to expression attacks of eyes closed, open mouth, sad, and angry, respectively. Figure 10(c1–c4) show the results of the 3D face key subjected to occlusion attacks of 12.5%, 25%, 37.5%, and 50%, respectively. As can be seen from Figure 10, although the 3D face key suffers from ±90° rotation attacks, different expression attacks, and 50% occlusion attacks, the corresponding FGS values are all higher than the preset threshold, resulting in successful decryption. Therefore, the proposed 3D face key exhibits strong robustness.

**Figure 11:**
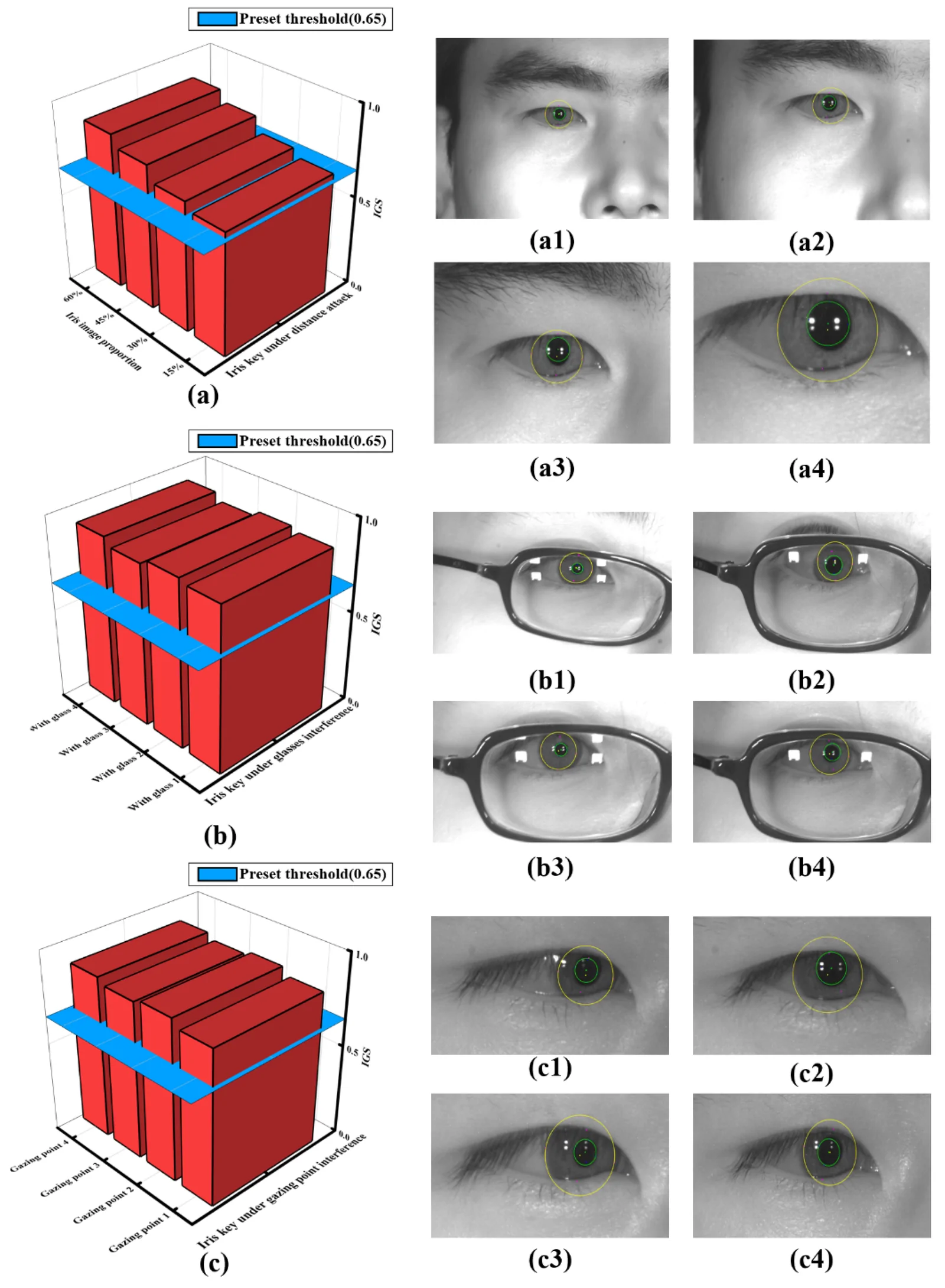
The robustness analysis results of the iris key in the proposed method. (**a**–**c**) the mapping relationship between the iris key and IGS under distance attack, glasses interference, and gazing point interference. (**a1**–**a4**) iris key subjected to iris key proportion of 15%, 30%, 45% and, 60%, respectively. (**b1**–**b4**) the iris key subjected to glasses interference of wear glasses 1, wear glasses 2, and wear glasses 3, respectively. (**c1**–**c4**) the iris key subjected to gazing point of gazing point 1, gazing point 2, and gazing point 3, respectively.

Next, Figure 11a–c display the mapping relationship between the iris and IGS under distance attack, glasses interference, and gazing point interference. It is worth noting that, to evaluate the robustness of the iris key against distance attack, the iris acquisition results at different collection distances are simulated by adjusting the proportion of the iris region within the captured image. Figure 11(a1–a4) show the results of the iris key subjected to iris key proportion of 15%, 30%, 45% and, 60%, respectively. Figure 11(b1–b4) show the results of the iris key subjected to glasses interference of wear glasses 1, wear glasses 2, and wear glasses 3, respectively. Figure 11(c1–c4) show the results of the iris key subjected to gazing point of gazing point 1, gazing point 2, and gazing point 3, respectively. As can be observed from Figure 11, although the iris key is subjected to distance attacks with an iris region proportion of 15%, various eyeglass interferences, and gazing point variations, the corresponding IGS values remain higher than the predefined threshold, resulting in successful decryption. This demonstrates that the iris key employed in the proposed method exhibits strong robustness against different types of attacks.

### 3.4. Host Image Robustness Analysis

**Figure 12:**
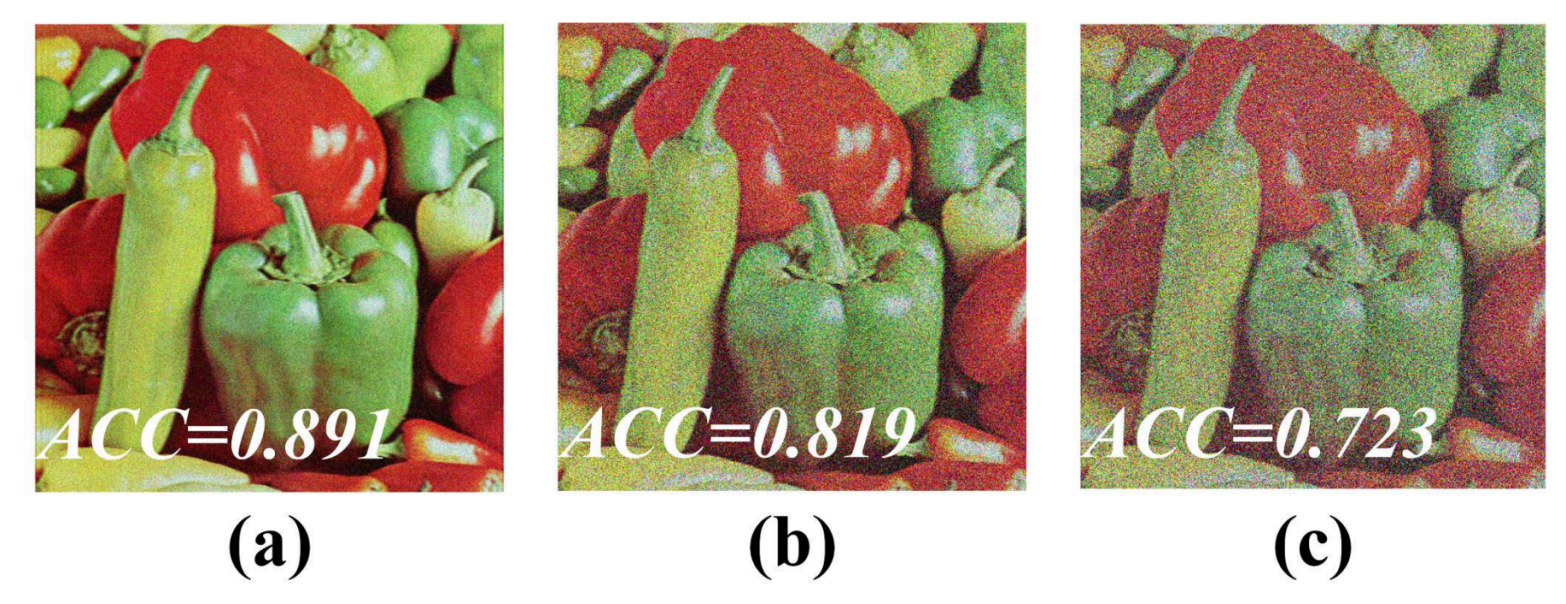
The Gaussian noise attack analysis results of the host image in the proposed method. (**a**–**c**) the decryption results corresponding to host image contaminated with noise strengths of 0.1, 0.5, and 1.0, respectively.

Moreover, the robustness performance of the host image under noise and occlusion attacks is evaluated. Specifically, Gaussian noise with various levels is introduced into the host image to assess its ability to withstand noise perturbations. The resulting noisy host image $H^{\Delta }(x,y)$ is described as:

(20) $$\;H^{\Delta }(x,y)=H(x,y)+\tau N$$

where N is the Gaussian noise with zero mean and 0.1 standard deviation, and the coefficient τ is the noise strength. Figure 12a–c display the noise-affected decryption results corresponding to host image contaminated with noise strengths of 0.1, 0.5 and 1.0, respectively.

**Figure 13:**
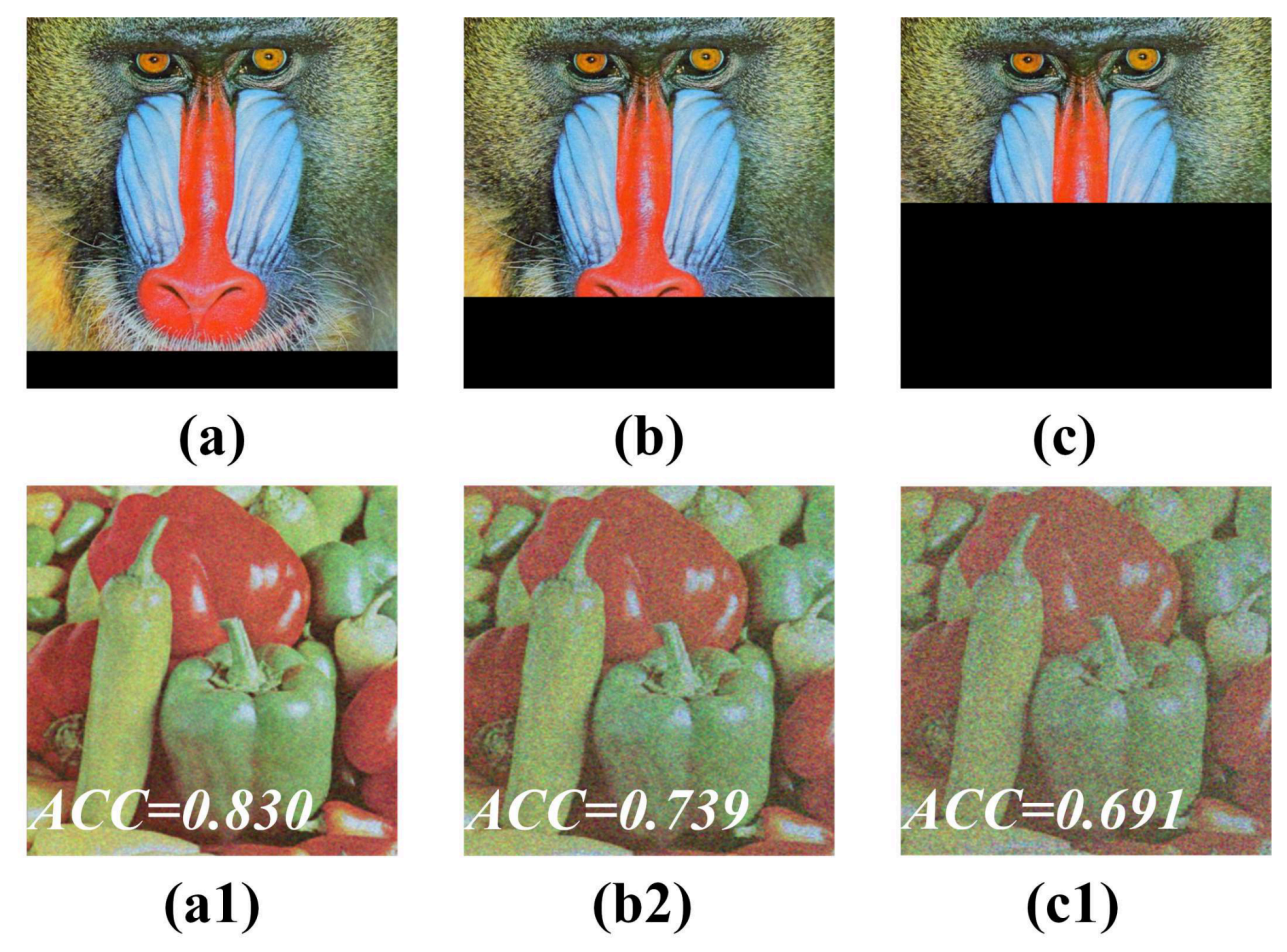
The occlusion attack analysis results of the host image in the proposed method. (**a**–**c**) the ciphertext with 12.5%,25%, and 50% occlusion; (**a1**,**b1**,**c1**) the decryption results corresponding to (**a**–**c**), respectively.

As shown in Figure 12, although the ACC value decreases with increasing intensity of Gaussian noise, the ACC value of the corresponding decryption results remains higher than 0.7 even under the maximum Gaussian noise intensity. Therefore, it can be demonstrated that the proposed method can effectively resist Gaussian noise attacks. In addition, in order to evaluate the ability of the host image against occlusion attacks, partial pixels of the host image were cropped. Figure 13a–c display the host image with 12.5%, 25% and 50% occlusion, respectively, while their corresponding decryption results are shown in Figure 13(a1,b1,c1). As shown in Figure 13, the ACC value of the decryption results decreases as the occlusion ratio increases. However, even when the occlusion ratio reaches 50%, the corresponding ACC value of the decryption results remains higher than 0.69. Therefore, it can be demonstrated that the proposed method can effectively resist occlusion attacks.

**Figure 14:**
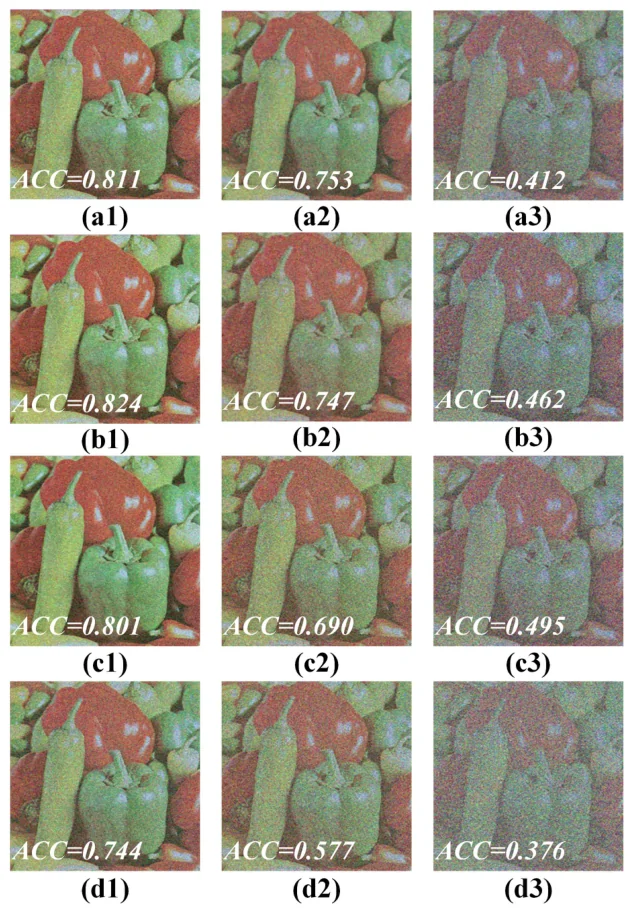
Extraction results of the original color watermark image under different attacks on the host image: (**a1**–**a3**) JPEG compression with compression levels of 50%, 70%, and 90%, respectively; (**b1**–**b3**) median filtering with kernel sizes of 3 × 3, 5 × 5, and 7 × 7, respectively; (**c1**–**c3**) average filtering with kernel sizes of 3 × 3, 5 × 5, and 7 × 7, respectively; and (**d1**–**d3**) rotation attacks with rotation angles of 3°, 5°, and 7°, respectively.

In addition, Figure 14(a1–a3) show the corresponding extraction results when the host image is subjected to JPEG compression with compression levels of 50%, 70%, and 90%, respectively. Figure 14(b1–b3) show the corresponding extraction results when the host image is subjected to median filtering with kernel sizes of 3 × 3, 5 × 5, and 7 × 7, respectively. Figure 14(c1–c3) present the corresponding extraction results under average filtering with kernel sizes of 3 × 3, 5 × 5, and 7 × 7, respectively. Figure 14(d1–d3) show the corresponding extraction results when the host image is subjected to rotation attacks with rotation angles of 3°, 5°, and 7°, respectively. As shown in Figure 14, the ACC of the extracted color watermark image generally decreases as the attack severity increases, indicating that stronger attacks have a greater impact on the extraction performance. Nevertheless, even under the most severe JPEG compression, median filtering, and average filtering attacks considered in these experiments, the ACC values of the extracted original color watermark images remain above 0.35. These results demonstrate that the host image employed in the proposed method exhibits effective robustness against common image-processing attacks, including JPEG compression, median filtering, average filtering, rotation, Gaussian noise, and occlusion attacks.

### 3.5. Comparative Analysis

First, to quantitatively compare the computational complexity of the proposed method with those reported in Refs. [29,30,31], Table 2 lists the parameters used in the simulation and the adopted configuration of the computing platform.

As shown in Table 3, although the digital key space of the proposed method is smaller than that of some existing optical zero-watermarking methods based on a single biometric key, the proposed method combines two independent biometric keys, namely the 3D face key and the iris key. The use of these bimodal biometric keys substantially increases the cost and complexity for unauthorized attackers to obtain sensitive information. Furthermore, it should be noted that the structured light phase mask (SPM) coupled in the proposed bimodal biometric chaotic structured light phase masks (BCSPMs) is constructed using relatively simple optical elements, i.e., the radial Hilbert mask (RHM) and the Fresnel zone plate (FZP). Accordingly, more complex SPMs with additional optical parameters will be explored in future work to further enlarge the key space and enhance the security of the proposed method. On the other hand, due to the adoption of the relatively complex bimodal biometric high-order feature extraction network (BBHEN), the proposed method requires slightly more computational time than the methods reported in Refs. [29,30,31] during the zero-watermark generation and extraction processes. The processing times for zero-watermark generation and zero-watermark extraction are only 2.24 s and 2.64 s, respectively. Although the incorporation of BBHEN introduces additional computational overhead, this trade-off is acceptable because the enhanced high-order feature extraction capability substantially improves the robustness and reliability of the biometric keys under various attacks. Therefore, the proposed method achieves a favorable balance between computational efficiency and biometric security.

## 4. Discussion and Conclusions

In this paper, we propose an optical color zero-watermarking construction method based on highly robust bimodal biometric keys and phase-shifting digital holography. By introducing highly robust 3D face and iris keys into optical watermarking, the proposed method achieves secure biometric authentication through a bimodal biometric high-order feature extraction network (BBHEN). Meanwhile, the combination of grating modulation, Fresnel-domain DRPE, phase-shifting digital holography, and Canny edge feature extraction enables the generation of a noise-like color zero-watermark image containing both host and watermark information. Benefiting from the extracted high-order biometric features and multi-dimensional digital keys, the proposed method provides a large key space, strong robustness against biometric key attacks, high invisibility, and excellent portability.

To demonstrate the feasibility of the proposed method, a series of computational simulations are conducted. The results indicate that the proposed method exhibits high feasibility and security. The enhanced security is mainly attributed to the large digital key space provided by the highly sensitive optical parameters and the additional identity verification mechanism provided by the bimodal biometric features. In particular, the portable 3D face key demonstrates strong robustness against rotation, expression, and occlusion attacks, while the iris key exhibits strong robustness against distance variations, glasses interference, and gaze point interference, verifying the practicality and reliability of the employed biometric keys. Furthermore, the resistance of the proposed method against various attacks is evaluated, and the results confirm its strong robustness against noise and occlusion attacks. Therefore, the proposed method has the following three main advantages over Refs. [29,30,31,32,33,34]: (1) Enhancement of biometric authentication security and robustness: Refs. [29,30,31,32,33,34] represent recently reported optical watermarking methods based on biometric keys. Although these studies successfully introduced biometric information into optical watermarking systems, some methods employ only a single biometric key and therefore provide only one biometric authentication mechanism. Moreover, although several methods adopt bimodal biometric keys, their robustness against various attacks remains insufficient. In contrast, the proposed bimodal biometric high-order feature extraction network (BBHEN) effectively extracts high-order features from both the iris key and 3D face key. The results demonstrate that both biometric keys maintain reliable authentication under the tested attacks. Furthermore, the proposed serial bimodal authentication mechanism requires both biometric modalities to satisfy the authentication criterion, thereby enhancing authentication security while maintaining the robustness of individual biometric keys under the tested attacks.

(2) Capability of color watermark image processing: Furthermore, most of the watermark images employed in Refs. [29,30,31,32,33,34] are grayscale images, which limits their applicability for processing color images containing independent R, G, and B components. In the proposed method, a grating modulation-based encryption fusion image generation technique is introduced, enabling the original color watermark image to be efficiently encoded into a noise-like zero-watermark image. Therefore, the proposed scheme can effectively handle color watermark images while maintaining high security and concealment.

(3) Expansion of key space and enhancement of security: In addition, to further enlarge the key space, the phase masks used in the DRPE process are not directly generated by random functions but are constructed by coupling the biometric information of the encryption user with the structured light phase mask (SPM). Since the SPM contains highly sensitive optical parameters, such as topological charge, focal length, and wavelength, small variations in these parameters can lead to significant changes in the generated phase mask. Consequently, the key space and security level of the proposed method are further enhanced.

In addition, the proposed bimodal authentication mechanism does not regard the iris and 3D face features as additional cryptographic key bits. However, the two biometric modalities serve as independent identity verification factors for controlling access to the decryption process. Specifically, successful authentication requires both the iris similarity score (IGS), the 3D face similarity score (FGS), and their fused similarity score (BBGS) to simultaneously satisfy the predefined threshold. Therefore, a high similarity score from one biometric modality cannot compensate for authentication failure in the other modality. This serial bimodal authentication strategy increases the difficulty of unauthorized biometric forgery and further enhances the security of the proposed system.

It should be noted that the present study is primarily based on computational simulations, in which the optical propagation, phase modulation, and phase-shifting digital holographic processes are numerically modeled using Python 3.9.0and MATLAB2021b. Although the simulation results demonstrate the feasibility, security, and robustness of the proposed method, a corresponding physical optical system has not yet been experimentally constructed and validated. Therefore, the absence of experimental verification under realistic optical conditions represents an important limitation of the current work. In future studies, a physical optical platform incorporating the required spatial light modulators and other optical components will be established to experimentally evaluate the proposed method and further investigate its practical feasibility and robustness under realistic optical conditions. It should also be acknowledged that dedicated physical prosthetic spoofing or presentation-attack experiments have not been conducted in the present study. The 3D face and iris keys used in this work are obtained from the publicly available FaceScape [36] and CASIA-IrisV4 [37] datasets, respectively, which do not provide physical prosthetic biometric samples for such evaluations. Therefore, the resistance of the proposed method to real-world prosthetic spoofing attacks cannot yet be fully established based on the current numerical simulations. Nevertheless, since the proposed authentication scheme employs both 3D face and iris keys, an attacker attempting to bypass the authentication system through prosthetic spoofing would need to simultaneously fabricate valid prosthetic samples for both biometric modalities. Compared with unimodal biometric authentication, this requirement substantially increases the difficulty, cost, and operational complexity of a successful spoofing attack.

In future work, more advanced spatial filtering strategies will be investigated to further reduce the loss of high-frequency information and improve the reconstruction quality of the extracted color watermark image. Meanwhile, more advanced biometric features, such as voiceprint and finger vein characteristics, will be incorporated into the proposed optical color zero-watermarking construction method to further enhance its practicality and adaptability. In addition, more complex structured light phase masks with higher-dimensional optical parameters and richer phase distributions will be explored and introduced into the proposed framework to further enlarge the key space and enhance the overall security of the proposed method. Advanced optical encryption technologies, including ptychographic imaging and metasurface holography, will also be explored and integrated into the proposed framework to expand its encryption capabilities and improve its security performance. These efforts are expected to further enhance the reconstruction performance, key space, security, and application potential of the proposed method in secure optical information processing and protection.

## Figures and Tables

**Table 1:** Key space data for each digital key.

Digital Key	Sensitivity	Value Range	Key Space	Total Key Space
$Z_{1}$	0.66 mm	0~2^15^	≈2^16^	≈2^93^
$Z_{2}$	0.63 mm	0~2^15^	≈2^16^	
$Z_{3}$	0.64 mm	0~2^15^	≈2^16^	
λ	0.72 nm	0~2^15^	≈2^15^	
f	0.80 mm	0~2^15^	≈2^15^	
ρ	0.77	0~2^15^	≈2^15^	

**Table 2:** The parameters used in the simulation and the adopted configuration of computing platform.

The parameters used in the simulation	Pixel pitch of each plaintext image	8 μm
	Wavelength of light λ	632.8 nm
	First diffraction distance $Z_{1}$	140 mm
	Second diffraction distance $Z_{2}$	150 mm
	Third diffraction distance $Z_{3}$	160 mm
	Focal length f	40 mm
	phase shift $\varphi_{\chi }$	0, *π*/2, and *π*
	Topological charge number ρ	3
The adopted configuration of computing platform	Central processing unit	i7-13700K
	Random access memory	40 GB
	General processing unit	RTX4070

**Table 3:** Comparison results between the proposed method and recently reported biometric key-based zero-watermarking methods.

Subjects		The Method in Ref. [29]	The Method in Ref. [30]	The Method in Ref. [31]	The Proposed Method
Watermark image type		Gray image	Gray image	Gray image	Color image
Number of biometric keys		1	2	1	2
Face key robustness	Face key type	2D data	2D data	2D data	3D data
	Rotation attack	±15°	±15°	±15°	±90°
	Expressions attack	Two Expressions	Two Expressions	Two Expressions	Four Expressions
	Occlusion attack	Unable to resist	Unable to resist	Unable to resist	50%
Iris key robustness	Distance attack	None	None	None	Effective
	Glasses interference attack	None	None	None	Effective
	Gazing point interference attack	None	None	None	Effective
Security	Key type	Digital key and 2D face key	Digital key and 2D facekey and fingerprint key	Digital key and 2D face key	Digital key and 3D face key and Iris key
	Digital key space	≈2^160^	≈2^62^	≈2^250^	≈2^98^
Computational complexity	Zero-watermark generation process	0.630 s	0.726 s	0.621 s	2.24 s
	Zero-watermark extraction process	0.611 s	0.676 s	0.631 s	2.64 s
Host image robustness	Occlusion attack	Range: 50%	Range: 50%	Range: 50%	Range: 50%
	Gaussian noise attack	Strength: 1.0	Strength: 1.0	Strength: 1.0	Strength: 1.0
	JPEG compression attack	Compression level: 90%	Compression level: 90%	Compression level: 90%	Compression level: 90%
	Median filtering attack	Size: 7 × 7	Size: 7 × 7	Size: 7 × 7	Size: 7 × 7
	Average filtering attack	Size: 7 × 7	Size: 7 × 7	Size: 7 × 7	Size: 7 × 7
	Rotation attack	Angle: 7°	Angle: 7°	Angle: 7°	Angle: 7°

## Data Availability

Data underlying the results presented in this paper are not publicly available at this time but may be obtained from the authors upon reasonable request.
